# Artificial intelligence in rheumatology and paediatric rheumatology: insights from an international survey by EMEUNET

**DOI:** 10.1016/j.ero.2026.03.001

**Published:** 2026-04-03

**Authors:** Saverio La Bella, Ana Isabel Rebollo-Giménez, Krystel Aouad, Latika Gupta, Tue Kragstrup, Deniz Bayraktar, Thomas Davergne, Davide Cangelosi, Thomas Hügle, Johannes Knitza, Nicolino Ruperto, Vincenzo Venerito, Wilson Bautista-Molano, Dzifa Dey, Abdellah El Maghraoui, Ihsane Hmamouchi, Alison M. Hoens, Linda C. Li, Angela Migowa, Erin Treemarcki, Nelly Ziade, Alessandra Alongi, Diego Benavent

**Affiliations:** 1 Department of Medicine and Surgery, University of Milano-Bicocca, Monza, Italy; 2 Paediatric Rheumatology INternational Trials Organisation (PRINTO), Monza, Italy; 3 Hospital General Universitario Gregorio Marañón, Health Research Institute Gregorio Marañón (IiSGM), Madrid, Spain; 4 Department of Rheumatology, Saint George Hospital University Medical Center, Saint George University of Beirut, Beirut, Lebanon; 5 Department of Rheumatology, Royal Wolverhampton Hospitals NHS Trust, Wolverhampton, UK; 6 School of Infection, Inflammation and Immunology, College of Medicine and Health, Birmingham, UK; 7 Francis Crick Institute, London, UK; 8 Department of Biomedicine, Aarhus University, Aarhus, Denmark; 9 Department of Rheumatology, Medical Diagnostic Center, Hospitals in Central Denmark Region, Silkeborg, Denmark; 10 Department of Molecular Medicine, Aarhus University Hospital, Aarhus, Denmark; 11 Department of Physiotherapy and Rehabilitation, Faculty of Health Sciences, Izmir Katip Celebi University, Izmir, Türkiye; 12 Department of Physical Therapy, Faculty of Medicine, University of Iceland, Reykjavik, Iceland; 13 Arthritis Research Canada, Vancouver, BC, Canada; 14 Université Paris Cité and Université Sorbonne Paris Nord, Inserm, INRAE, Center for Research in Epidemiology and Statistics (CRESS), Paris, France; 15 Clinical Bioinformatics Unit, IRCCS Istituto Giannina Gaslini, Genoa, Italy; 16 Department of Rheumatology, University Hospital Lausanne (CHUV) and University of Lausanne, Lausanne, Switzerland; 17 Institute for Digital Medicine, Philipps-Universität Marburg, Marburg, Germany; 18 Fondazione IRCCS San Gerardo dei Tintori, Reumatologia Pediatrica, PRINTO, Monza, Italy; 19 Department of Precision and Regenerative Medicine and Ionian Area (DiMePRe-J), University of Bari “Aldo Moro”, Bari, Italy; 20 Rheumatology Section, University Hospital Fundacion Santa Fe de Bogotá, School of Medicine Universidad El Bosque, Bogotá, Colombia; 21 Rheumatology Unit, Department of Medicine and Therapeutics, Korle Bu Teaching Hospital, University of Ghana Medical School, Accra, Ghana; 22 Rheumatology Office, Mohamed V University, Rabat, Morocco; 23 Health Sciences Research Center (CReSS), Faculty of Medicine, International University of Rabat, Rabat, Morocco; 24 Department of Physical Therapy, University of British Columbia, Vancouver, BC, Canada; 25 Aga Khan University Medical College East Africa, Nairobi, Kenya; 26 Department of Pediatrics, Division of Pediatric Rheumatology, University of Utah Health, Salt Lake City, UT, USA; 27 Rheumatology Department, Saint Joseph University, Hotel-Dieu de France Hospital, Beirut, Lebanon; 28 Rheumatology Department, Hospital Universitari de Bellvitge, L’Hospitalet de Llobregat, Barcelona, Spain

## Abstract

**Objectives:**

Artificial intelligence (AI) is revolutionising medicine. The aim of this study was to detail its use, opinions, knowledge, and concerns in rheumatology and paediatric rheumatology.

**Methods:**

A web-based survey open to all professionals working in the field was developed by the Emerging EULAR Network (EMEUNET) and disseminated between March and July 2025 in collaboration with other international rheumatology societies (AFLAR, ArLAR, CARRA, PAFLAR, PANLAR). The survey was divided into 4 sections: (i) participants’ characteristics, (ii) AI use and applications, (iii) opinions and knowledge, and (iv) concerns, needs, and expectations.

**Results:**

Overall, 461 responses were collected from 59 countries. Respondents were mostly physicians who completed their training (316, 68.7%) and were based in Europe (170, 36.9%). Most participants (397, 86.7%) used AI for medical purposes, especially large language models (385, 83.7%) for grammar correction and brainstorming. Although there was broad optimism about its use (366, 79.6%), self-reported practical skills were predominantly basic or still in development (346, 75.1%), and knowledge was rarely defined as strong or expert-level (63, 13.7%). Concerns focused on ethics (314, 69%), lack of trust (316, 69.5%), and insufficient training (270, 59.3%). Disparities emerged across geographic regions in use, knowledge, and practical skills.

**Conclusions:**

AI is widely used and positively perceived in rheumatology, despite limited knowledge and practical skills, and regional disparities. Addressing gaps in ethics, transparency, and insufficient training through targeted education and implementation strategies will be essential to ensure an equitable and effective integration into clinical and research practice.

**WHAT IS ALREADY KNOWN ON THIS TOPIC**

• There is a growing interest in artificial intelligence (AI) in rheumatology and paediatric rheumatology for both clinical practice and research. In parallel, national and international societies are increasingly engaging in discussions and forming working groups on the topic.
• Although the use of AI tools is widespread and publications on their applications are steadily increasing, no broad international data are available on how physicians and other professionals in the field perceive and use these technologies.

**WHAT THIS STUDY ADDS**

• This study provides the first broad international overview of how physicians and other professionals in rheumatology perceive, understand, and use AI.
• It documents a widespread and predominantly positive attitude and optimism towards AI, despite generally limited basic knowledge and skills in using AI in clinical practice.
• This survey identifies key gaps in ethical awareness, practical skills, and training, as well as geographic disparities, highlighting priority areas for targeted education and structured implementation strategies.

**HOW THIS STUDY MIGHT AFFECT RESEARCH, PRACTICE OR POLICY**

• The identification of geographic and professional disparities may provide actionable evidence for policymakers and societies to promote equitable access, standardised guidance, and responsible adoption of systems powered by AI across clinical practice and research.
• By understanding real-world use patterns, perceptions of AI, and unmet needs, this study can inform the design of evidence-based policies to promote the safe and transparent integration of AI technologies.
• This study can guide the development of targeted training programmes and structured implementation pathways.

Alt-text: Unlabelled box dummy alt text

## INTRODUCTION

There is a growing use of artificial intelligence (AI) in the field of rheumatology [[1], [2], [3], [4]]. Among its applications, large language models (LLMs) are increasingly adopted for daily clinical practice and research tasks, allowing physicians and other health professionals in rheumatology (HPRs) to access knowledge more rapidly and improve overall efficiency [[5], [6], [7]]. Although harmful hallucinations and confabulations from LLMs should still be taken into account, relevant progress has been made recently, ranging from the adoption of retrieval-augmented generation approaches to the emergence of free online decision-support platforms [[8], [9], [10], [11], [12]]. Beyond LLMs, sophisticated AI systems are used to improve diagnostic and management strategies, reduce repetitive workloads, assist in data analysis, and predict disease progression [[13], [14], [15], [16]]. On this basis, international societies have included dedicated sessions on AI in their congresses to promote interactive discussions and better disseminate information on the latest advancements. In the field of rheumatology, both the European Alliance of Associations for Rheumatology (EULAR) and the Paediatric Rheumatology European Society have likewise allocated considerable attention in their most recent conferences [17,18]. In 2020, the EULAR Points to Consider for the use of big data in rheumatic and musculoskeletal diseases (RMDs) were published, providing foundational guidance for the responsible application of large-scale data resources in rheumatology [19]. Given the rapid methodological advances and the expanding role of AI in health research, a new EULAR task force is being developed to provide updated recommendations and points to consider for the use of big data in RMDs, on this occasion, including the use of AI [20]. Within this framework, the EMerging EULAR NETwork (EMEUNET) represents a unique platform to capture the perspectives of the rheumatology community. As the official EULAR committee for early-career rheumatologists and researchers, EMEUNET has grown into one of the largest networks of early-career clinicians and researchers in rheumatology worldwide [21]. Over recent years, EMEUNET has conducted multiple surveys addressing key topics, with a special interest in professional development and innovation in rheumatology [[22], [23], [24], [25], [26], [27]]. Building on this experience and aligned with EULAR’s strategic direction, EMEUNET has now initiated dedicated work on AI. This exploratory survey was developed through EMEUNET to better understand (i) current patterns of AI use in clinical practice and research, (ii) self-reported knowledge and skills, (iii) attitudes, concerns and perceived needs, and (iv) whether these domains differ across world regions.

## METHODS

### Survey development methodology

Methods are reported according to the Checklist for Reporting Results of Internet E-Surveys (CHERRIES) [28]. The development of the questionnaire followed a consensus-based methodology. All members involved in the survey development had either a background or a specific interest in AI. An EMEUNET internal core group first defined the scope and work plan of the project (SLB, AIR-G, KA, LG, TK, and D Benavent); to ensure broad participation among HPRs, members of the EULAR HPR community were involved at this stage as part of the group (D Bayraktar and TD). Based on these insights, an initial draft of the survey was prepared in English and then, on discussion within the EMEUNET group, was extended to external experts in consensus strategies and AI in rheumatology (DC, TH, JK, NR, and VV). This step was necessary to ensure an appropriate level of expertise, ultimately improving the scientific accuracy of the survey. A second draft was developed within the extended methodology group through multidisciplinary discussion and shared with early-career representatives of other international rheumatology societies to expand perspectives and enhance international applicability. Representatives from the African League of Associations for Rheumatology, the Pan Arab Society of Rheumatic Diseases, the Childhood Arthritis and Rheumatology Research Alliance, the Paediatric Society of the African League Against Rheumatism, and the Pan-American League of Associations for Rheumatology participated in the development and dissemination of the survey. All comments were integrated through iterative revisions (Supplementary Fig S1). Overall, the web-based survey consisted of 29 questions, including multiple-choice items, single-answer items, and Likert-scale responses; some variables were collected both as Likert-type scores and as nominal categories (questions and answers are available as Supplementary Material S1). The survey was approved by consensus. No formal psychometric validation was undertaken, as the primary aim of the study was exploratory. Questions were divided into 4 sections: (i) participants’ characteristics, (ii) use and applications, (iii) opinion and knowledge, and (iv) concerns, needs, and expectations. In this study, knowledge referred to the theoretical understanding of AI concepts, skills referred to the practical ability to use AI tools effectively, and literacy referred to the combination of knowledge and skills that enabled safe, informed, and critical use of AI in clinical and research practice.

The survey was voluntary and specifically designed for individuals working in the field of rheumatology and paediatric rheumatology, with no exclusion criteria. Response rates were not displayed to participants during survey completion, and no incentives were provided for participation. Before accessing the questionnaire, all participants were required to read an information sheet and provide explicit written consent. No ethical approval was required, as the survey did not collect any health-related data and was not accessible to patients. The online survey was launched via Google Forms and disseminated in March 2025 through social media channels. No cookies, IP checks, log file analyses, or registration-based mechanisms were used to prevent or identify duplicate entries, to maximise participant privacy. The survey was open-access, and no technical filtering of duplicate responses was applied. The data collected were stored on secure platforms with access restricted by password protection and 2-factor authentication. Data collection remained open until July 10, 2025.

### Statistical analysis

Descriptive and summary statistics were calculated based on the total number of responses per question. Continuous data were reported as mean and SD or median and 1st to 3rd quartiles. Categorical data were presented as counts and percentages. Associations between the ordinal and continuous variables of interest were examined using Spearman’s rank correlation, with coefficients interpreted according to Cohen’s conventional thresholds [29]. Countries were classified by continent according to the United Nations M49 geographical classification. For descriptive purposes, the Americas were further disaggregated into North America and South America to better reflect differences in the availability, implementation, and use of AI (Supplementary Materials S2 and S3). Given the exploratory aim of the study, results are reported descriptively, without formal statistical inference. No imputation was performed; analyses were conducted using all available responses for each survey item. Analyses were performed using the R software environment for statistical computing and graphics (R Foundation for Statistical Computing, Vienna, Austria; version 4.2).

## RESULTS

### Characteristics of the survey participants

**Figure 1:**
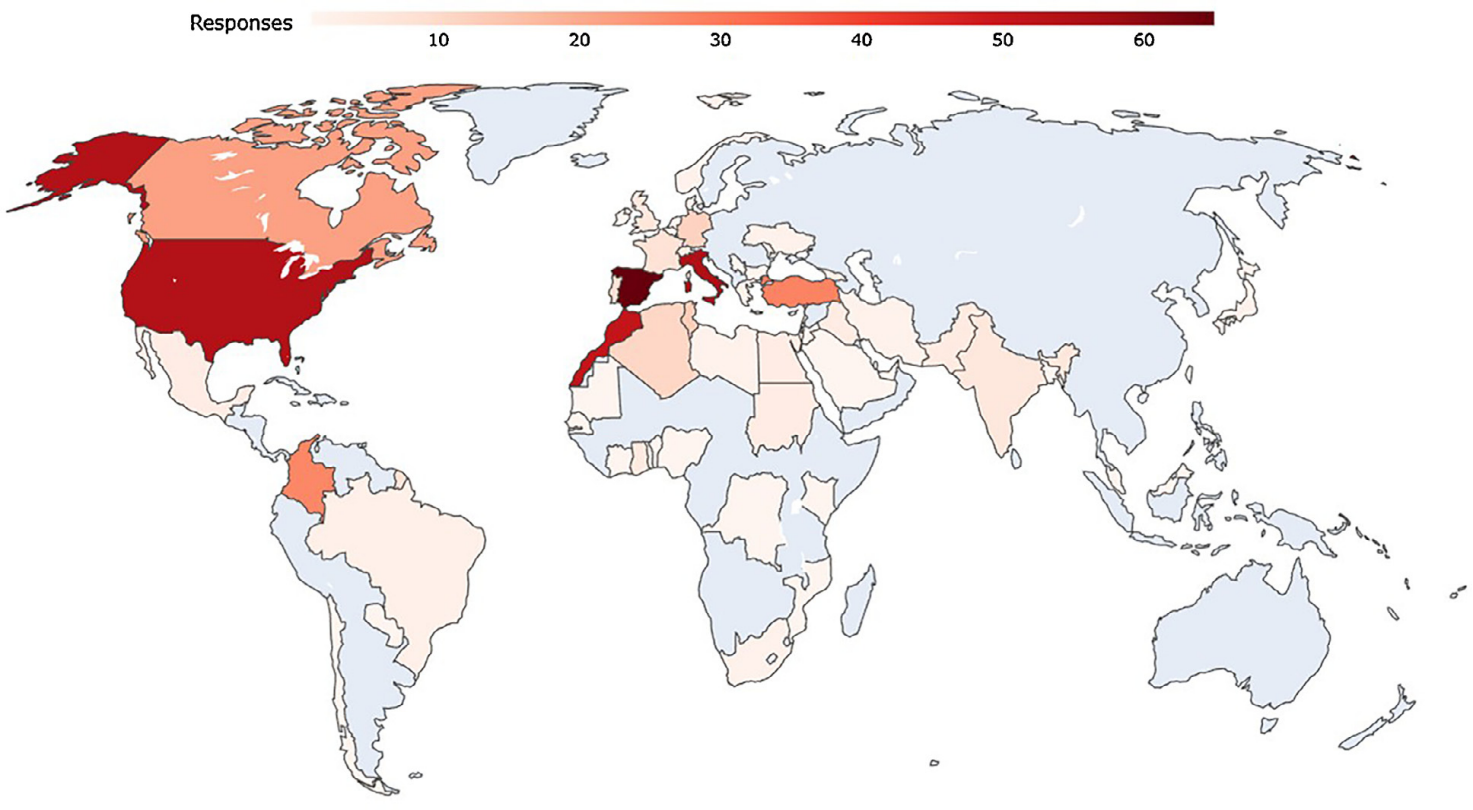
Distribution of survey respondents by country of work. The survey collected 461 responses from 59 countries. Europe accounted for the largest proportion of participants (37%), followed by the Americas (25%), Africa (22%), and Asia (16%). No respondents were from Oceania. The most represented countries were Spain (59, 13%), Italy (55, 12.1%), United States (54, 11.9%), and Morocco (52, 11.4%).

Overall, the survey collected 461 responses from 59 countries (Fig. 1). Respondents were predominantly female (293, 63.6%) and had a median age of 39.5 years (Q1-Q3: 33-49). Spain (59, 13%) was the most represented country, followed by Italy (55, 12.1%), the United States (54, 11.9%), and Morocco (52, 11.4%) (Supplementary Material S4). Most participants were from Europe (170, 36.9%), and the Americas (112, 24.3%). Physicians comprised the majority of respondents (409, 88.7%), most of whom were consultants who had completed their training (316, 68.5%). Within HPRs, physical therapists (24, 5.2%) were the most represented category. Rheumatology was the main work field (317, 68.8%), while fewer participants worked in paediatric rheumatology (128, 27.8%) or in both fields (14, 3%). Respondents most frequently spent the majority of their work in clinical activity (374, 81.1%) and worked in a university hospital/research institute (321, 69.9%) (Table 1).

**Table 1:** Characteristics of the participants (N = 461)

Variable	Count (percentage)
Gender	
Female	293 (63.6)
Male	167 (36.2)
Missing	1 (0.2)
Age (y; median and 1st-3rd quartiles), (n = 454)	39.5 (33-49)
Main field of interest	
Rheumatology	317 (68.8)
Paediatric rheumatology	128 (27.8)
Equally both	14 (3.0)
Missing	2 (0.4)
Main occupation	
Clinical practice	374 (81.1)
Research	87 (18.9)
Current occupation	
Medical doctor (specialist)	316 (68.5)
Medical doctor (trainee)	83 (18)
Physical therapist	24 (5.2)
Researcher	12 (2.6)
Psychologist	4 (0.9)
Nurse	4 (0.9)
Research assistant	4 (0.9)
Data analyst/scientist	3 (0.7)
Occupational therapist	3 (0.7)
Other	7 (1.5)
Missing	1 (0.2)
Current occupation, by category	
Physician	409 (88.7)
Health professionals	38 (8.2)
Other	14 (3.0)
Workplace	
University hospital/research institute	321 (69.9)
Private practice	65 (14.1)
Public healthcare centre/nonuniversity hospital	64 (13.9)
Primary care setting	6 (1.3)
Other	3 (0.7)
Missing	2 (0.4)
Continent	
Africa	102 (22.1)
Americas	112 (24.3)
Asia	71 (15.4)
Europe	170 (36.9)
Missing	6 (1.3)
Region	
East Asia and Pacific	4 (0.9)
Europe and Central Asia	201 (43.6)
Latin America and Caribbean	36 (7.8)
Middle East and North Africa	106 (23)
North America	76 (16.5)
South Asia	12 (2.6)
Sub-Saharan Africa	20 (4.3)
Missing	6 (1.3)

Categories with less than 3 responses have been grouped as ‘other’. Country details are provided as Supplementary Material S4.

### Use and applications

The survey revealed broad use of AI, with 397 participants (86.7%) reporting having used it in clinical practice or research; among them, 112 respondents (24.5%) used AI systems daily or almost daily, while only 61 respondents (13.3%) had never used AI for medical purposes. The first use occurred in 2024 for 216 participants (47.2%), while for 83 respondents (18.1%) it occurred between 2022 and 2023 and for 80 participants (17.5%) in 2025.

Among the various AI models, 385 participants (83.7%) used LLMs, and 130 (28.3%) used other AI tools to retrieve medical knowledge. A minority used AI platforms for data analysis (68, 14.8%), assisted diagnosis (45, 9.8%), or decision support (28, 6.1%). Some respondents used AI for administrative purposes such as scheduling and documentation (79, 17.2%). LLMs were used in medical settings for text correction and grammar refinement by 296 participants (64.5%), and for brainstorming and summarising articles or texts by 235 (51.2%) and 217 (47.3%) participants. Moreover, 174 respondents (37.9%) used LLMs for nonmedical purposes like fun or entertainment.

More than 1 in 3 participants (162, 35.3%) had paid for a subscription plan, with some respondents holding an active subscription (138, 30.1%). Most participants experienced the efficiency of AI through time saved in routine tasks (151, 33%) or through personal experience and feedback from colleagues or patients (154, 33.6%). Although 145 respondents (31.5%) reported preliminary discussions about potential integration of AI in the workplace, only 104 (22.6%) indicated that 1 or more AI tools had actually been implemented.

**Figure 2:**
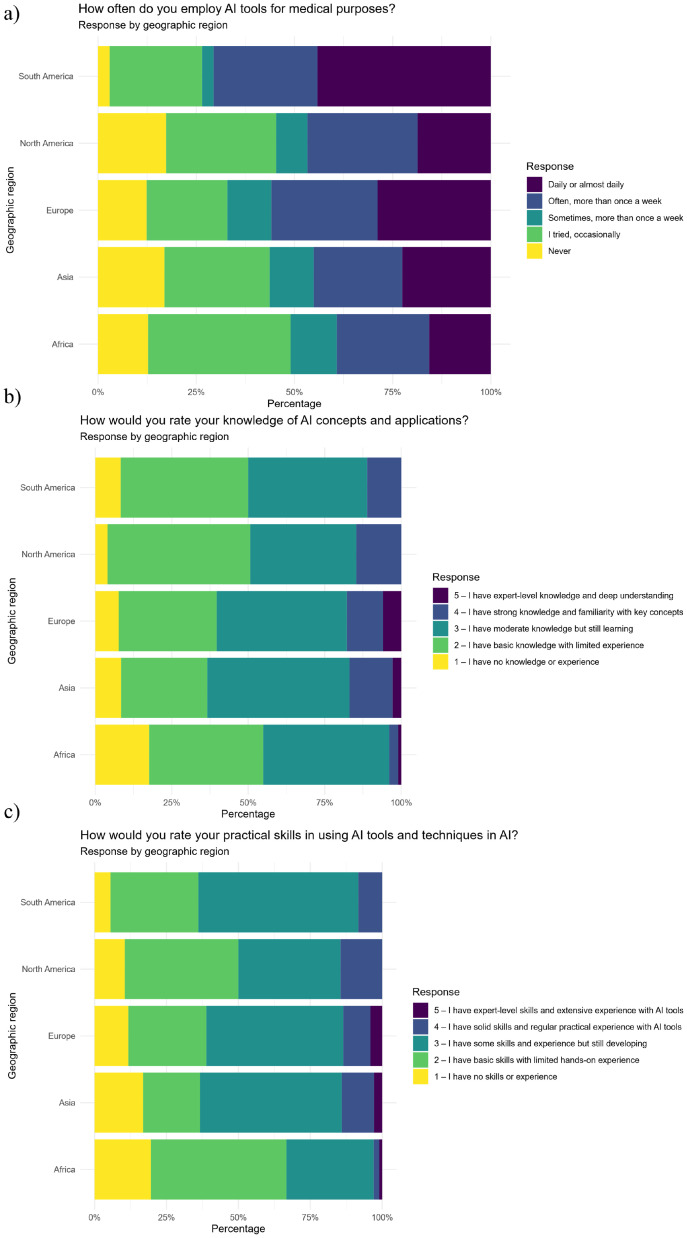
Regional differences in AI use, knowledge, and practical skills. A) Respondents from South America reported more frequent medical use of AI than those from Africa, Asia, and North America; Europe also reported higher use than Africa. B) AI knowledge differed across regions, with higher scores in Europe and Asia compared with Africa. C) Practical AI skills were lowest among respondents from Africa, with significantly higher scores in Europe, Asia, South America, and North America.

There was some variability across regions in the use and applications of AI tools. Respondents from South America (median 4 [Q1-Q3: 2-5]) used AI for medical purposes more frequently than those from Africa (median 3 [Q1-Q3: 2-4]), Asia (median 3 [Q1-Q3: 2-4]), and North America (median 3 [Q1-Q3: 2-4]). Respondents from Europe used AI in medical settings (median 4 [Q1-Q3: 2-5]) more frequently than those from Africa (median 3 [Q1-Q3: 2-4]) (Fig. 2A). Respondents from North America had the highest level of AI implementation in their workplace (47% when already implemented, and 78% when also considering exploratory projects) (Supplementary Material S5).

### Opinion and knowledge

Participants expressed predominantly favourable attitudes towards AI’s efficacy and safety. Most of them declared themselves to be ‘optimistic with caution’ (274, 59.6%) or ‘positive and optimistic’ (92, 20%). Scepticism was uncommon (23, 5%) and strong criticism was rare (2, 0.4%). Although there was substantial enthusiasm, proficiency was generally reported as limited: knowledge was most often moderate (189, 41.2%) or basic (164, 35.7%), while respondents with strong (50, 10.9%) and expert-level understanding (13, 2.8%) were less frequent. Practical skills showed a similar pattern, predominantly still in development (195, 42.3%) or basic (151, 32.8%); expert-level skills were reported in a minority of cases (10, 2.2%). Despite the low number of nonphysicians among respondents, including data analysts and bioinformaticians, this group reported the highest self-declared skills and knowledge. Instead, physicians were the category with the most positive opinion of AI (Supplementary Material S6). Similar to use and applications, there was some variability across regions in the knowledge and practical skills of AI tools. Specifically, participants from Europe (median 3 [Q1-Q3: 2-3]) had better knowledge of AI than those from Africa (median 2 [Q1-Q3: 2-3]). Similarly, respondents from Asia (median 3 [Q1-Q3: 2-3]) had better knowledge of AI than those from Africa (median 2 [Q1-Q3: 2-3]) (Fig. 2B). Practical skills in using AI solutions and techniques were lower in participants from Africa (median 2 [Q1-Q3: 2-3]) than those from Europe (median 3 [Q1-Q3: 2-3]), Asia (median 3 [Q1-Q3: 2-3]), South America (median 3 [Q1-Q3: 2-3]), and North America (median 3 [Q1-Q3: 2-3]) (Fig. 2C).

**Figure 3:**
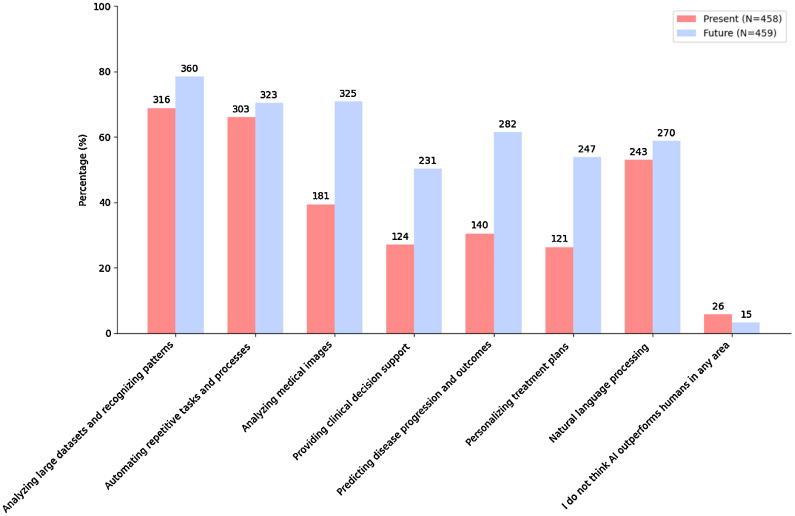
Present and future areas where artificial intelligence is expected to outperform humans. The most frequently cited capabilities were analysing large datasets and recognizing patterns, automating repetitive tasks and processes, and natural language processing. Respondents anticipated an increase in AI performance across all areas in the future, and only a small number of participants believed that AI does not currently outperform, and will not outperform, humans in any area. Multiple answers were allowed.

At present, only a small minority of participants did not indicate any domain of superiority over humans (26, 5.7%). The leading areas of current superiority were analysing large datasets and recognising patterns (316, 69%), and automating repetitive tasks (303, 66.2%). Future superiority is expected to be higher across all domains, particularly data analysis and pattern recognition (360, 78.4%), with the strongest expected gains in predicting disease progression (282, 61.4%) (Fig. 3). From an ethical perspective, most respondents favoured robust oversight of AI to ensure safety and effectiveness. Indeed, 260 participants (56.9%) supported heavy regulation, alongside 171 respondents (37.4%) who supported calibrated regulation so as not to hinder innovation; only a minority supported minimal regulation (19, 4.2%), or no oversight (7, 1.5%). AI literacy was recognised as a key skill, rated as very or extremely important by 192 (41.7%) and 169 (36.7%) respondents, respectively; none rated it as not important. In the research setting, more than half of respondents supported LLMs use for drafting emails and correcting language (343, 74.7%), copyediting (326, 71%), brainstorming (278, 60.6%), and assisting in accessing references (276, 60.1%). Almost half of the respondents supported the use of LLMs in assisting article writing, such as delivering part of the text (210, 45.8%).

A strong positive correlation emerged between practical AI skills and AI knowledge (r = 0.79). AI use was moderately to strongly positively correlated with a positive opinion about AI efficacy and safety (r = 0.45), AI knowledge (r = 0.51), and practical AI skills (r = 0.59). Lastly, AI knowledge (r = −0.19) and practical AI skills (r = −0.23) were weakly negatively correlated with age (Supplementary Fig S2).

### Concerns, needs, and expectations

The medical domains where AI systems were perceived to have the greatest impact were mainly research (352, 76.7%), administrative tasks (321, 69.9%), and diagnosis (299, 65.1%). Substantial expectations were also reported for professional communication tasks, such as e-mail correspondence, manuscript review, and editorial feedback (269, 58.6%), and for patient-facing communication. There were also relevant expectations for clinical decision support (207, 45.1%) and medical education (206, 44.9%). Focusing on research, expectations regarding areas where AI is believed to have the greatest impact now or in the coming years were especially strong for accelerating the analysis and synthesis of scientific information (375, 83.3%), facilitating reference management (280, 62.2%), and optimising publishing and peer-review processes (262, 58.2%). Concerns were mainly due to reliability, ethics, and implementation capacity. The most frequent challenge was a lack of trust in AI-provided information due to misinformation, confabulation, and misleading outputs (316, 69.5%), closely followed by ethical issues related to data privacy and accountability (314, 69%). Skill gaps were relevant issues, with several participants declaring insufficient training or understanding (270, 59.3%) and limited interpretability of AI outputs (188, 41.3%). Practical barriers consisted of regulatory and legal uncertainty (176, 38.7%), inadequate integration with existing systems (165, 36.3%), and lack of informatics resources (127, 27.9%) (Table 2).

**Table 2:** Survey responses on artificial intelligence among physicians and other professionals working in rheumatology and paediatric rheumatology

Questions and responses	Count (percentage)
Use and applications	
How often do you employ AI tools for medical purposes? (n = 458)	
5 - Daily or almost daily	112 (24.5)
4 - Often, more than once a week	117 (25.5)
3 - I tried, occasionally	121 (26.4)
2 - Sometimes, more than once a week	47 (10.3)
1 - Never	61 (13.3)
When did you start using AI in your work? (n = 458)	
In the current year (2025)	80 (17.5)
Since last year (2024)	216 (47.2)
In the past 2 to 3 years (2022-2023)	83 (18.1)
More than 3 years ago (up to 2021)	16 (3.5)
I have not used it yet	63 (13.8)
Which AI tools or platforms do you use for medical purposes (multiple answers allowed) (N = 460)	
Large language models	385 (83.7)
AI-based diagnostic tools	45 (9.8)
AI-assisted decision-support systems	28 (6.1)
AI to look up medical knowledge	130 (28.3)
AI for data analysis	68 (14.8)
AI for administrative purposes	79 (17.2)
I do not use any AI tools	53 (11.5)
Other	12 (2.4)
What are your purposes of using large language models? (multiple answers allowed) (N = 459)	
Activities not related to research, such as fun and entertainment	174 (37.9)
Brainstorming ideas for projects, research, or discussion	235 (51.2)
Text correction and English language refinement	296 (64.5)
Assistance in medical education	144 (31.4)
Summarising papers, articles, or texts	217 (47.3)
Assistance in paper writing	183 (39.9)
Help with grant applications	76 (16.6)
Help in writing codes	80 (17.4)
Data analysis	90 (19.6)
Assistance in creating images	132 (28.8)
I have never used large language models	48 (10.5)
Other	11 (2.2)
How do you assess the effectiveness of AI tools in your practice? (N = 458)	
Through personal observation or feedback from colleagues or patients	154 (33.6)
Using specific performance metrics or outcomes	33 (7.2)
By the time saved in routine tasks	151 (33)
I do not assess the effectiveness	117 (25.5)
Other	3 (0.6)
Have you ever had a subscription plan for AI tools? (N = 459)	
Yes, I have a subscription plan	138 (30.1)
Yes, I have had a subscription plan	24 (5.2)
No	297 (64.7)
In 2025, do you know if your workplace has explored, implemented, or considered using artificial intelligence in clinical practice and research? (multiple answers allowed) (N = 460)	
Yes, 1 or more AI tools have been implemented and are actively used in clinical practice and/or research	104 (22.6)
Yes, pilot studies or exploratory projects have been conducted to assess its feasibility and usefulness	73 (15.9)
Yes, concrete plans for implementation are being discussed for the near future, but no formal adoption has taken place yet	65 (14.1)
No, but there is interest and preliminary discussions about its potential integration in the future	145 (31.5)
No, and do not seem to be any plans or interest in incorporating AI at this time	69 (15)
I do not know, I am not aware of any AI-related initiatives in my workplace	62 (13.5)
Opinion and knowledge	
How would you rate your opinion about AI efficacy and safety? (N = 460)	
5 - Positive and optimistic	92 (20)
4 - Optimistic with caution	274 (59.6)
3 - Neutral or undecided	69 (15)
2 - Sceptical or concerned	23 (5)
1 - Negative or critical	2 (0.4)
How would you rate your knowledge of AI concepts and applications? (N = 459)	
5 - I have expert-level knowledge and deep understanding	13 (2.8)
4 - I have strong knowledge and familiarity with key concepts	50 (10.9)
3 - I have moderate knowledge but still learning	189 (41.2)
2 - I have basic knowledge with limited experience	164 (35.7)
1 - I have no knowledge or experience	43 (9.4)
How would you rate your practical skills in using AI tools and techniques in AI? (N = 461)	
5 - I have expert-level skills and extensive experience with AI tools	10 (2.2)
4 - I have solid skills and regular practical experience with AI tools	42 (9.1)
3 - I have some skills and experience but still developing	195 (42.3)
2 - I have basic skills with limited hands-on experience	151 (32.8)
1- I have no skills or experience	63 (13.7)
How do you think AI should be regulated in the medical field? (N = 457)	
AI applications should be heavily regulated to ensure safety and effectiveness	260 (56.9)
Some regulation is needed, but not too stringent to hinder innovation	171 (37.4)
AI should be minimally regulated to foster development and adoption	19 (4.2)
AI should be free to develop without any governmental or institutional oversight	7 (1.5)
How important do you think it is for health professionals to have AI literacy? (N = 460)	
Extremely important: AI literacy is essential for all health professionals, both now and in the future	169 (36.7)
Very important: AI literacy is becoming increasingly crucial for health professionals	192 (41.7)
Moderately important: AI literacy is helpful but not necessarily essential for all health professionals	93 (20.2)
Not important: AI literacy is not necessary for health professionals	0 (0)
Unsure: I am uncertain about the importance of AI literacy for health professionals	6 (1.3)
Do you support the use of large language models for any of the following purposes in research settings to reduce time consumption and improve the process? (multiple answers allowed) (N = 459)	
Drafting emails and correcting language	343 (74.7)
Assisting authors in brainstorming ideas when writing a paper	278 (60.6)
Assisting authors in writing a paper (delivering part of the text)	210 (45.8)
Assisting authors in copyediting and correcting a paper (grammar, spelling, language editing)	326 (71)
Assisting reviewers in evaluating papers and peer-review process	185 (40.3)
Assisting editors in prescreening papers after submission (underscoring strong and weak points)	180 (39.2)
Assisting authors in having an easier access to references (automatic search, summaries)	276 (60.1)
Assisting in creating figures or visual aids for papers	266 (58)
I have no experience with research	28 (6.1)
I do not support the use of large language models for any research purpose	9 (2)
Other	2 (0.4)
Which of the following AI models or tools are you familiar with (ie, have basic knowledge of) (multiple answers allowed) (N = 453)	
Supervised machine learning models	86 (19)
Unsupervised machine learning models	67 (14.8)
Large language models	355 (78.4)
Natural language processing techniques	108 (23.8)
Imaging tools	92 (20.3)
Deep learning models	56 (12.4)
AI-based decision-support systems	71 (15.7)
Predictive analytics models	54 (11.9)
None of these	63 (13.9)
Concerns, needs, and expectations	
In which medical areas do you believe AI tools could have the most impact? (multiple answers allowed) (N = 459)	
Diagnosis	299 (65.1)
Treatment planning	168 (36.6)
Clinical decision support	207 (45.1)
Patient monitoring	228 (49.7)
Patient communication	230 (50.1)
Communication	269 (58.6)
Research	352 (76.7)
Administration	321 (69.9)
Medical education	206 (44.9)
Other	5 (1)
In which areas of research do you believe AI has the greatest impact now or will have in the coming years? (multiple answers allowed) (N = 450)	
Analysis and synthesis of scientific information	375 (83.3)
Optimisation of the publishing and peer-review process	262 (58.2)
Improvement in citation and reference management	280 (62.2)
Experimental design and optimisation	176 (39.1)
Facilitation of scientific collaboration	191 (42.4)
Other	7 (1.4)
What are the main challenges or concerns you have when using AI tools in your practice? (multiple answers allowed) (N = 455)	
Lack of trust in AI decisions (eg, misleading or false outputs, misinformation)	316 (69.5)
Lack of interpretability of AI outputs (eg, unclear processes, features)	188 (41.3)
Insufficient training or understanding of AI tools	270 (59.3)
Ethical concerns (eg, data privacy, accountability)	314 (69)
Lack of integration with existing systems	165 (36.3)
Lack of resources and informatic support	127 (27.9)
Regulatory and legal issues	176 (38.7)
Other	13 (2.6)
Would you recommend using AI tools in medical practice to your colleagues? (N = 458)	
Yes, strongly recommend	135 (29.5)
Yes, with some reservations	257 (56.1)
No, I would not recommend	10 (2.2)
I don’t have enough experience to say	56 (12.2)

AI, artificial intelligence.

Multiple answers were allowed for several items.

## DISCUSSION

This study offers a wide overview of how AI is being adopted in clinical and research activities in rheumatology and paediatric rheumatology. We found that more than 8 out of 10 respondents reported using AI tools for clinical or research purposes, with a growing trend over the past years. Moreover, the majority of participants were using AI platforms for medical tasks more than once a week at the time of the survey, and a notable proportion of active users had subscribed to paid AI platforms, indicating the perceived utility and value of these tools.

Although LLMs were widely used, mainly for research activities, other tools designed for data analysis, diagnostic, or decision support were generally less familiar to the survey participants. As expected, research emerged as the leading medical area where AI tools could have the strongest impact according to the respondents, surpassing administration and diagnosis. This perception could be primarily related to the opportunities provided by an easy capacity of access, analysis, and synthesis of scientific information, a benefit that has also been reported from the patients’ perspective [30]. Around 4 out of 10 respondents also supported the use of LLMs for improving and boosting the peer-review process by actively assisting editors and reviewers, opening a discussion that will probably become increasingly important. Interestingly, respondents did not indicate using AI for tasks like conducting research, participant recruitment (eg, creating recruitment materials), or data collection. This pattern points to the early phase of AI integration in these domains, with practices still developing.

Our insights reveal a dual landscape within the scientific community: while attitudes are predominantly positive and open, technical knowledge and practical skills remain limited. As already documented in previous studies, physicians generally express enthusiasm and acceptance towards AI, with responses largely characterised by ‘cautious optimism’ and ‘positive and optimistic’ perspectives [31,32]. However, in our survey, this optimism is not correlated with an acknowledged solid competency base or practical skills: the majority of respondents reported only ‘basic’ or ‘moderate’ knowledge, and a similar proportion described their practical skills as ‘developing’ or ‘basic’. Similar to previous reports, the discrepancy between enthusiasm and knowledge raises concerns about potential overconfidence when using AI systems, as these tools may not be fully understood, increasing the risk of amplified errors or biases in the absence of structured training [33]. This issue becomes even more evident when considering regional differences in AI-related knowledge and practical skills, with disparities in both domains mainly observed in respondents from African countries. However, despite a higher implementation reported by participants from North America, no substantial variability emerged across continents regarding the perceived importance of AI literacy, suggesting a tendency towards unequal distribution of opportunities rather than differences in digital orientation or goals. In this context, previous reports indicate that knowledge, use, and attitudes towards clinical AI vary between countries; for example, a survey conducted in late 2024 reported higher AI use in China compared with other countries, and differences in optimism about its future impact across regions [34]. These findings suggest that regional factors, such as access to training and infrastructure, may contribute to regional variations in AI competency and adoption. Moreover, recent evaluations of LLM-generated responses to clinical questions in paediatric rheumatology showed that terminology and thematic emphasis can also differ between regions, highlighting that regional context may influence AI-generated medical content and emphasising the need for critical appraisal and adaptation in diverse healthcare settings [35].

On the other hand, clinicians and researchers recognise AI’s transformative potential but remain aware of associated risks, as this optimism was balanced by substantial concerns regarding reliability, data governance, and the limited interpretability of current systems. Furthermore, although preliminary discussions about workplace AI integration are common, actual implementation remains less frequent. This highlights a gap between positive perception, personal use of AI tools, and concrete organisational uptake, potentially attributable to structural inertia or practical challenges in AI deployment. Strong scepticism or criticism is uncommon, suggesting that cultural resistance, traditionally viewed as a major barrier to emerging technological adoption, is not a leading factor in this context. Governance mechanisms that ensure safety and effectiveness while avoiding approaches that might hinder innovation will be important and are being developed. For example, the American Heart Association has proposed pragmatic governance and monitoring principles intended to safeguard safety, effectiveness, and equity in the adoption of AI in healthcare, illustrating how professional societies can play a central role in guiding responsible implementation [36].

AI literacy was rated as very or extremely important by almost all respondents, in line with previous surveys, expert opinions, and position papers, underscoring a growing recognition that technical and critical understanding of AI is an essential professional competency [32,[37], [38], [39], [40], [41], [42]]. In this direction, a systematic review has recently highlighted the growing demand for digital and technical skills, although ambiguity persists regarding how these skills should be integrated into medical training [43]. Alongside a possible overconfidence in AI systems, the frequent identification of gaps in training underscores a perceived need for targeted educational initiatives and the development of tools with more transparent reasoning processes [[44], [45], [46], [47]]. All these findings highlight the need to promote responsible AI literacy and improve equitable access to training, ensuring that enthusiasm translates into competent and safe use in clinical and research practice. These reflections provide an opportunity for international societies to address this training gap and enhance their educational courses and resources on AI.

There are some limitations in the study. Selection bias may have occurred through the use of EMEUNET and online dissemination channels, potentially leading to an over-representation of respondents from Europe and of early adopters of AI. However, to mitigate this potential bias, we also involved additional international networks that contributed to disseminating the survey more broadly. Self-selection bias cannot be excluded, as individuals with more favourable or less critical attitudes towards AI might have been more likely to participate in the survey. The relatively small number of nonphysician participants and the absence of patient perspectives might also reduce the generalisability of the findings. Further limitations are the absence of a formal validation and the lack of mechanisms to prevent duplicate responses. Although this approach was chosen to prioritise privacy, a possible impact on data integrity cannot be entirely excluded. Data were self-reported, including measures of knowledge, skills, and AI usage; therefore, participants’ responses may have been influenced by social desirability bias, potentially leading to over-reporting of positive engagement. The survey did not explore the potential risk of deskilling, including reduced clinical reasoning and critical appraisal skills associated with prolonged exposure to AI-supported decision-making.

In conclusion, our findings underscore the expanding role of AI in accelerating the evolution of clinical and research practices within rheumatology and paediatric rheumatology. Difficulties may persist in awareness, access, and practical implementation, highlighting the need for coordinated efforts to ensure equitable integration of these emerging technologies. Improving responsible AI literacy and promoting transparent and reliable systems will be essential steps in bridging the gap between individual enthusiasm and structured adoption. This survey could inform future educational initiatives and guide ethical and implementation-oriented strategies for the sustainable adoption of AI in the field.

## Appendix Supplementary materials

Image, application 1

Image, application 2

Image, application 3

Image, application 4

Image, application 5

Image, application 6

Image, application 7

Image, application 8

## Footnotes

Supplementary material associated with this article can be found in the online version at doi:10.1016/j.ero.2026.03.001.
