# Supplementary material for: Artificial intelligence in rheumatology and paediatric rheumatology: insights from an international survey by EMEUNET
Source: EULAR Rheumatol Open. 2026 Apr 3;2(2):100153. doi: 10.1016/j.ero.2026.03.001 (PMC13425164; doi:10.1016/j.ero.2026.03.001)

**Supplementary Figure S1. Workflow of the survey development and dissemination process within EMEUNET.** The figure illustrates the sequential steps from initial definition and conceptualization by the EMEUNET methodology core group, through drafting, external review, and extension to international early-career representatives, to the final approval and dissemination of the web-based survey via EMEUNET and other societies channels.


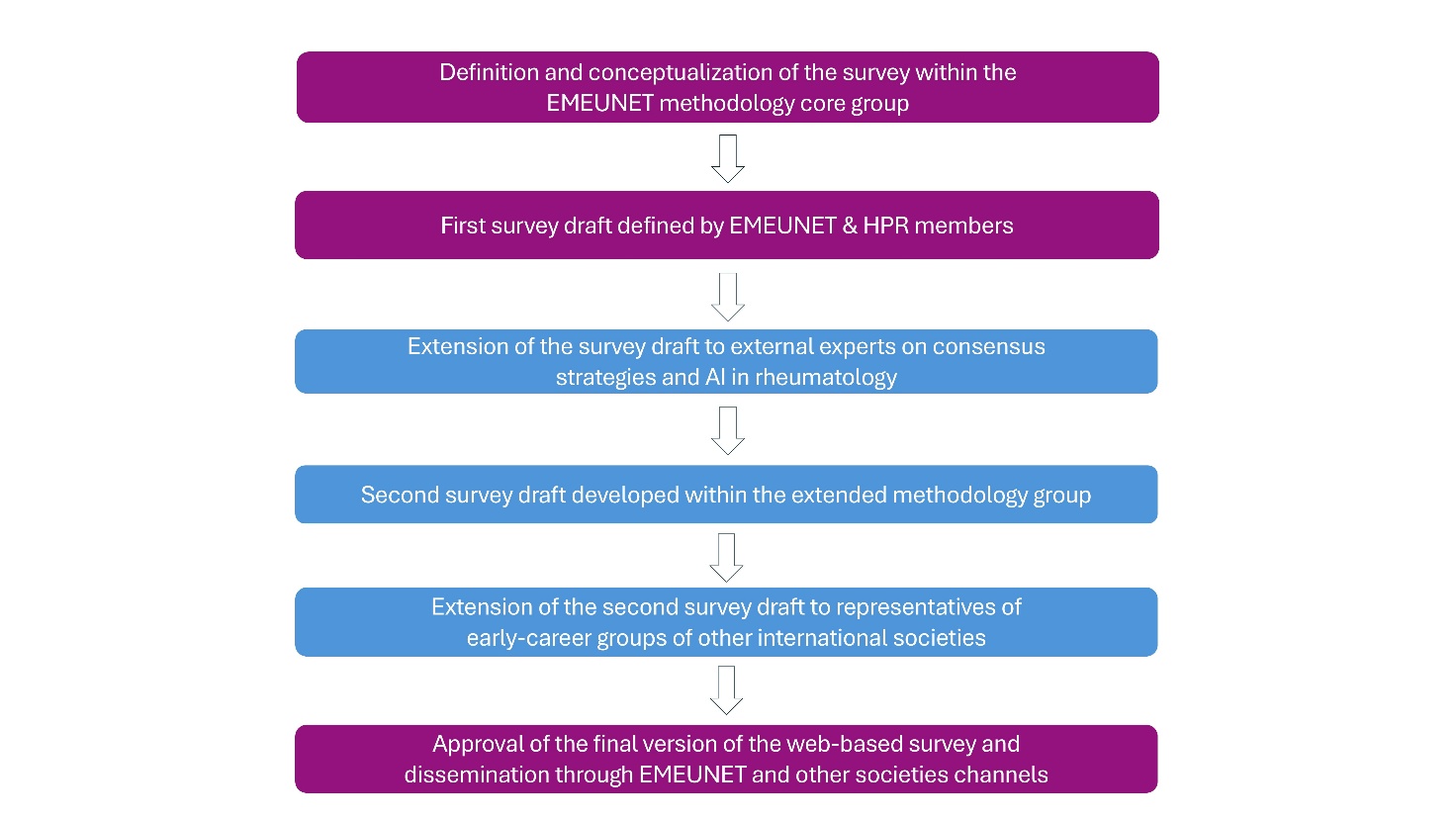

Supplement: Supplementary file 1 [file mmc1.docx]
