# Supplementary material for: Artificial intelligence in rheumatology and paediatric rheumatology: insights from an international survey by EMEUNET
Source: EULAR Rheumatol Open. 2026 Apr 3;2(2):100153. doi: 10.1016/j.ero.2026.03.001 (PMC13425164; doi:10.1016/j.ero.2026.03.001)

**Supplementary Figure S2.**Spearman’s correlation matrix of age, AI use, AI efficacy and safety perception, AI knowledge, and practical AI skills. AI knowledge showed a strong positive correlation with practical AI skills (r = 0.79). AI use was moderately to strongly positively correlated with a positive opinion about AI efficacy and safety (r = 0.45), AI knowledge (r = 0.51), and practical AI skills (r = 0.59). Both AI knowledge (r = -0.19) and practical AI skills (r = -0.23) were weakly negatively correlated with age. Warmer colours indicate stronger positive correlations, while cooler colours indicate negative correlations.


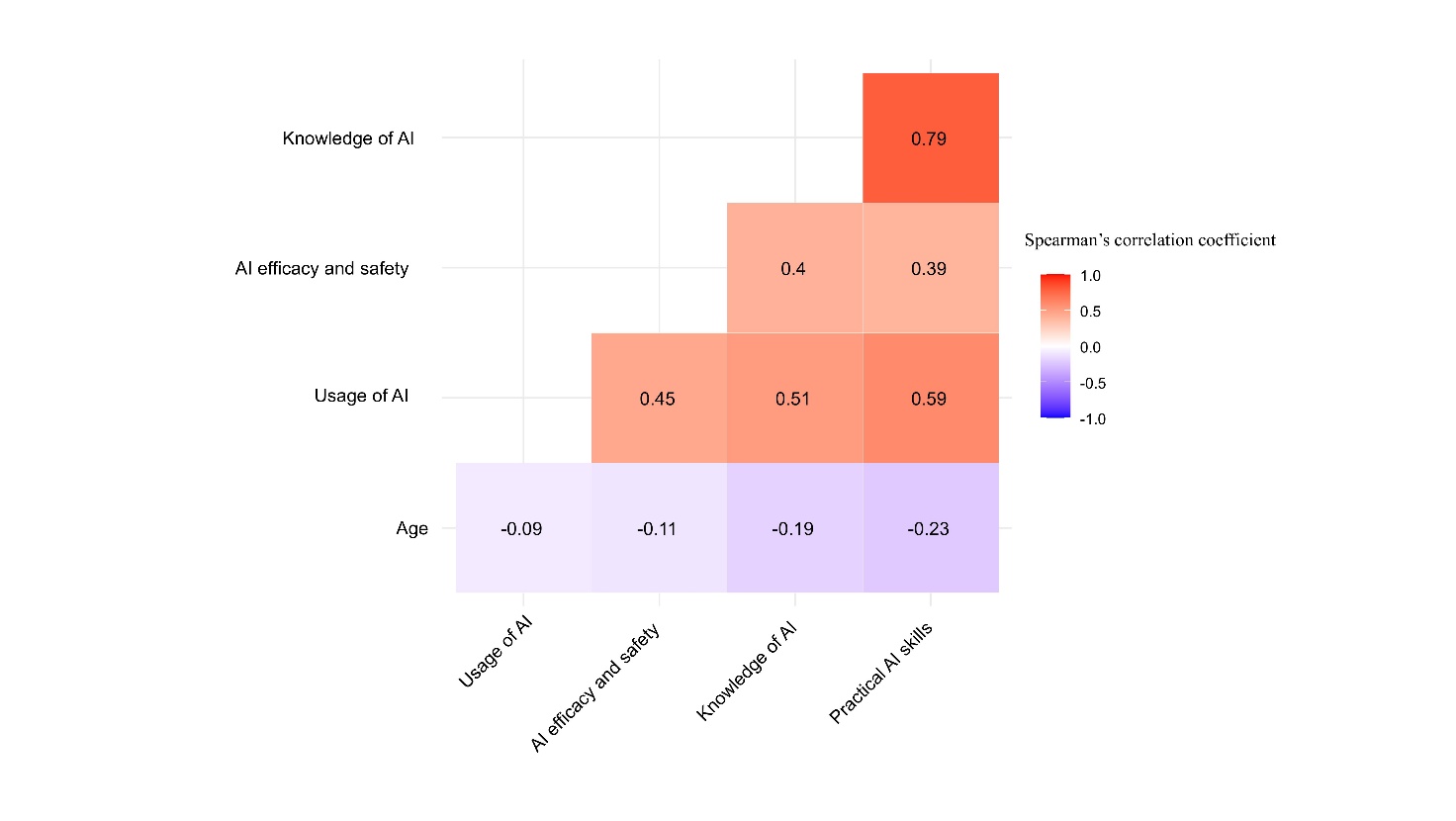

Supplement: Supplementary file 2 [file mmc2.docx]
