# Supplementary material for: Artificial intelligence in rheumatology and paediatric rheumatology: insights from an international survey by EMEUNET
Source: EULAR Rheumatol Open. 2026 Apr 3;2(2):100153. doi: 10.1016/j.ero.2026.03.001 (PMC13425164; doi:10.1016/j.ero.2026.03.001)
Supplement: Supplementary file 3 [file mmc3.docx]

**Supplementary Material S1.** Survey questions and answers

1. **Characteristics of the sample**

**How do you spend most of your time at work?**

- Clinical practice
- Research

**Which gender do you most identify with?**

- Female
- Male

**What is your age (in years)?**

**In which country do you work?**

**What is your current position?**

- Medical doctor - resident or fellow
- Medical doctor - specialist
- Nurse
- Physical therapist (PT)
- Occupational therapist (OT)
- Clinical psychologist
- Researcher
- Other (please specify)

**What is your main specialty field?**

- Rheumatology
- Pediatric rheumatology
- Other (please specify)

**Where do you primarily work?**

- Private practice (individual or group practice)
- Primary care setting
- Public healthcare center or non-university hospital
- University hospital or research institute
- Other (please specify)

**In 2025, do you know if your workplace has explored, implemented, or considered using artificial intelligence in clinical practice or research? (multiple answers allowed)**

- Yes, one or more AI tools have already been implemented and are actively used in clinical practice and/or research.
- Yes, pilot studies or exploratory projects have been conducted to assess its feasibility and usefulness.
- Yes, concrete plans for implementation are being discussed for the near future, but no formal adoption has taken place yet.
- No, but there is interest and preliminary discussions about its potential integration in the future.
- No, and there do not seem to be any plans or interest in incorporating AI at this time.
- I don’t know, I am not aware of any AI-related initiatives in my workplace.

1. **Usage and applications**

**How often do you employ AI tools for medical purposes?**

- Daily or almost daily
- Often, more than once a week
- Sometimes, more than once a month
- I tried, occasionally
- Never

**When did you start using AI in your work?**

- In the current year (2025)
- Since last year (2024-2025)
- In the past 2-3 years (2022-2023)
- More than 3 years ago (up to 2021)
- I have not used it yet

**Which AI tools or platforms do you use for medical purposes (multiple answers allowed)**

- Large language models
- AI-based diagnostic tools (e.g., imaging analysis, predictive analysis)
- AI-assisted decision support systems
- AI to look up medical knowledge
- AI for data analysis (e.g., code writing)
- AI for administrative purposes (e.g., scheduling, documentation)
- I do not use any AI tools
- Other (please specify)

**What are your purposes of using large language models? (multiple answers allowed)**

- Activities not related to research (fun, entertainment, etc.)
- Brainstorming ideas for projects, research, or discussion
- Text correction and English language refinement (papers, e-mails etc.)
- Assistance in medical education (e.g., Creating multiple-choice questions (MCQs), rubrics etc.)
- Summarizing papers, articles, or texts
- Assistance in paper writing
- Help with grant applications
- Help in writing codes (statistics, programming)
- Data analysis
- Assistance in creating images (e.g., graphs, visualizations)
- I have never used LLMs
- Other (please specify)

**How do you assess the effectiveness of AI tools in your practice?**

- Through personal observation or feedback from colleagues or patients
- Using specific performance metrics or outcomes
- By time saved in routine tasks
- I don’t assess the effectiveness
- Other (please specify)

**Have you ever had a subscription plan for AI tools?**

- Yes, I have a subscription plan
- Yes, I have had a subscription plan
- No

1. **Opinions and knowledge**

**How would you rate your opinion about AI efficacy and safety?**

- 5 – Positive and optimistic
- 4 – Optimistic with caution
- 3 – Neutral or undecided
- 2 – Skeptical or concerned
- 1 – Negative or critical

**How would you rate your knowledge of AI concepts and applications?**

- 5 – I have expert-level knowledge and deep understanding
- 4 – I have strong knowledge and familiarity with key concepts
- 3 – I have moderate knowledge but still learning
- 2 – I have basic knowledge with limited experience
- 1 – I have no knowledge or experience

**How would you rate your practical skills in using AI tools and techniques in AI?**

- 5 – I have expert-level skills and extensive experience with AI tools
- 4 – I have solid skills and regular practical experience with AI tools
- 3 – I have some skills and experience but still developing
- 2 – I have basic skills with limited hands-on experience
- 1 – I have no skills or experience

**In which areas do you think AI currently outperforms humans? (multiple answers allowed)**

- Analyzing large datasets and recognizing patterns (e.g., early disease detection, clustering of patient profiles)
- Automating repetitive tasks and processes (e.g., scheduling, data entry)
- Analyzing medical images (e.g., radiology, pathology slides)
- Providing clinical decision support for clinicians in complex cases (e.g., diagnostic tools, AI-assisted consultations)
- Predicting disease progression and outcomes using patient data (e.g., prognosis modeling)
- Personalizing treatment plans by integrating patient data (e.g., genomics, clinical history)
- Natural language processing (e.g., language translation, identifying positive and critical issues in research papers, sentiment analysis)
- I do not think AI outperforms humans in any area
- Others (please specify)

**In which areas do you think AI will outperform humans in the future? (multiple answers allowed)**

- Analyzing large datasets and recognizing patterns (e.g., early disease detection, clustering of patient profiles)
- Automating repetitive tasks and processes (e.g., scheduling, data entry)
- Analyzing medical images (e.g., radiology, pathology slides)
- Providing clinical decision support for clinicians in complex cases (e.g., diagnostic tools, AI-assisted consultations)
- Predicting disease progression and outcomes using patient data (e.g., prognosis modeling)
- Personalizing treatment plans by integrating patient data (e.g., genomics, clinical history)
- Natural language processing (e.g., language translation, identifying positive and critical issues in research papers, sentiment analysis)
- I do not think AI will outperform humans in any area
- Others (please specify)

**How do you think AI should be regulated in the medical field?**

- AI applications should be heavily regulated to ensure safety and effectiveness
- Some regulation is needed, but not too stringent to hinder innovation
- AI should be minimally regulated to foster development and adoption
- AI should be free to develop without any governmental or institutional oversight

**How important do you think it is for health professionals to have AI literacy?**

- Extremely important: AI literacy is essential for all health professionals, both now and in the future.
- Very important: AI literacy is becoming increasingly crucial for health professionals.
- Moderately important: AI literacy is helpful but not necessarily essential for all health professionals.
- Not important: AI literacy is not necessary for health professionals
- Unsure: I am uncertain about the importance of AI literacy for health professionals

**Do you support the use of large language models for any of the following purposes in research settings to reduce time consumption and improve the process? (multiple answers allowed)**

- Drafting e-mails and correcting language
- Assisting authors in brainstorming ideas when writing a paper
- Assisting authors in writing a paper (delivering part of the text)
- Assisting authors in copy-editing and correcting a paper (grammar spelling, language editing)
- Assisting reviewers in evaluating papers and peer-review process
- Assisting editors in pre-screening papers after submission (underscoring strong and weak points)
- Assisting authors in having an easier access to references (automatic search, summaries)
- Assisting in creating figures or visual aids for papers
- I have no experience with research
- I do not support the use of LLMs for any research purpose
- Other (please specify)

**Which of the following AI models or tools are you familiar with (i.e., have basic knowledge of) (multiple answers allowed)**

- Supervised machine learning models (e.g., random forest)
- Unsupervised machine learning models (e.g., clustering)
- Large language models (e.g., ChatGPT, Gemini, other chatbots)
- Natural language processing techniques (e.g., text analysis, clinical notes processing)
- Imaging tools (e.g., AI for medical imaging analysis)
- Deep learning models (e.g., convolutional neural networks)
- AI-based decision support systems (e.g., clinical decision support tools)
- Predictive analytics models (e.g., predicting disease progression or outcomes)
- None of these

1. **Concerns, needs, and hopes for AI implementation in clinical practice and research**

**In which medical areas do you believe AI tools could have the most impact? (multiple answers allowed)**

- Diagnosis (e.g., radiology, pathology)
- Treatment planning
- Clinical decision support
- Patient monitoring
- Patient communication (chatbot to answer simple questions)
- Communication (e.g., e-mails, paper review, editorial feedback)
- Research (e.g., data analysis, literature review, article summary)
- Administration (e.g., scheduling, patient records)
- Medical education
- Other (please specify)

**In which areas of research do you believe AI has the greatest impact now or will have in the coming years? (multiple answers allowed)**

- Analysis and synthesis of scientific information
- Optimization of the publishing and peer-review process
- Improvement in citation and reference management
- Experimental design and optimization
- Facilitation of scientific collaboration
- Other (please specify)

**What are the main challenges or concerns you have when using AI tools in your practice? (multiple answers allowed)**

- Lack of trust in AI decisions (e.g., misleading or false outputs, misinformation)
- Lack of interpretability of AI outputs (e.g., unclear processes, features)
- Insufficient training or understanding of AI tools
- Ethical concerns (e.g., data privacy, accountability)
- Lack of integration with existing systems
- Lack of resources and informatic support
- Regulatory and legal issues
- Other (please specify)

**Would you recommend using AI tools in medical practice to your colleagues?**

- Yes, strongly recommend
- Yes, with some reservations
- No, I would not recommend
- I don’t have enough experience to say
