# Supplementary material for: Artificial intelligence in rheumatology and paediatric rheumatology: insights from an international survey by EMEUNET
Source: EULAR Rheumatol Open. 2026 Apr 3;2(2):100153. doi: 10.1016/j.ero.2026.03.001 (PMC13425164; doi:10.1016/j.ero.2026.03.001)
Supplement: Supplementary file 4 [file mmc4.docx]

**Supplementary Material S2.** Countries grouped in continents (data on 455 out of 461).

| Africa (n=102) | North America (n=76) | Central and South America (n=36) | Asia (n=71) | Europe (n=170) |
| --- | --- | --- | --- | --- |
| Algeria | Canada | Brazil | Bahrain | Belgium |
| Benin | USA | Chile | Bangladesh | Bulgaria |
| Democratic Republic of The Congo |  | Colombia | Georgia | Denmark |
| Egypt |  | Mexico | India | France |
| Ghana |  | Paraguay | Iran | Germany |
| Ivory Coast |  |  | Iraq | Greece |
| Kenya |  |  | Israel | Ireland |
| Libya |  |  | Japan | Italy |
| Mauritania |  |  | Jordan | Moldova |
| Morocco |  |  | Lebanon | Norway |
| Mozambique |  |  | Malaysia | Portugal |
| Nigeria |  |  | Pakistan | Serbia |
| Senegal |  |  | Palestine | Spain |
| South Africa |  |  | Qatar | Switzerland |
| Sudan |  |  | Saudi Arabia | The Netherlands |
| Togo |  |  | Taiwan | UK |
| Tunisia |  |  | Türkiye | Ukraine |
|  |  |  | United Arab Emirates |  |
