# Supplementary material for: Artificial intelligence in rheumatology and paediatric rheumatology: insights from an international survey by EMEUNET
Source: EULAR Rheumatol Open. 2026 Apr 3;2(2):100153. doi: 10.1016/j.ero.2026.03.001 (PMC13425164; doi:10.1016/j.ero.2026.03.001)
Supplement: Supplementary file 5 [file mmc5.docx]

**Supplementary Material S3.** Countries grouped in regions (data on 455 out of 461).

| East Asia and Pacific | Europe and Central Asia | Latin America and Caribbean | Middle East and North Africa | North America | South Asia | Sub-Saharan Africa |
| --- | --- | --- | --- | --- | --- | --- |
| Japan | Belgium | Brazil | Algeria | Canada | Bangladesh | Benin |
| Malaysia | Bulgaria | Chile | Bahrain | Usa | India | Democratic Republic of The Congo |
| Taiwan | Denmark | Colombia | Egypt |  | Pakistan | Ghana |
|  | France | Mexico | Iran |  |  | Ivory Coast |
|  | Georgia | Paraguay | Iraq |  |  | Kenya |
|  | Germany |  | Israel |  |  | Mauritania |
|  | Greece |  | Jordan |  |  | Mozambique |
|  | Ireland |  | Lebanon |  |  | Nigeria |
|  | Italy |  | Lybia |  |  | Senegal |
|  | Moldova |  | Morocco |  |  | South Africa |
|  | Norway |  | Palestine |  |  | Sudan |
|  | Portugal |  | Qatar |  |  | Togo |
|  | Serbia |  | Saudi Arabia |  |  |  |
|  | Spain |  | Tunisia |  |  |  |
|  | Switzerland |  |  |  |  |  |
|  | The Netherlands |  | United Arab Emirates |  |  |  |
|  | Türkiye |  |  |  |  |  |
|  | UK |  |  |  |  |  |
|  | Ukraine |  |  |  |  |  |
