# Supplementary material for: Artificial intelligence in rheumatology and paediatric rheumatology: insights from an international survey by EMEUNET
Source: EULAR Rheumatol Open. 2026 Apr 3;2(2):100153. doi: 10.1016/j.ero.2026.03.001 (PMC13425164; doi:10.1016/j.ero.2026.03.001)
Supplement: Supplementary file 6 [file mmc6.docx]

**Supplementary Material S4.**  Survey participants went from 59 different countries (data on 455 out of 461).

| Country | Count | Percentage |
| --- | --- | --- |
| Spain | 59 | 12,97 |
| Italy | 55 | 12,09 |
| USA | 54 | 11,87 |
| Morocco | 52 | 11,43 |
| Colombia | 27 | 5,93 |
| Türkiye | 27 | 5,93 |
| Canada | 22 | 4,84 |
| Tunisia | 13 | 2,86 |
| Germany | 12 | 2,64 |
| Algeria | 11 | 2,42 |
| Portugal | 9 | 1,98 |
| France | 8 | 1,76 |
| Lebanon | 7 | 1,54 |
| India | 6 | 1,32 |
| UK | 6 | 1,32 |
| Ghana | 5 | 1,1 |
| Pakistan | 5 | 1,1 |
| Egypt | 4 | 0,88 |
| Georgia | 4 | 0,88 |
| Iraq | 4 | 0,88 |
| Mexico | 4 | 0,88 |
| Sudan | 4 | 0,88 |
| Switzerland | 4 | 0,88 |
| Brazil | 3 | 0,66 |
| Denmark | 3 | 0,66 |
| Israel | 3 | 0,66 |
| Belgium | 2 | 0,44 |
| Greece | 2 | 0,44 |
| Iran | 2 | 0,44 |
| Japan | 2 | 0,44 |
| Jordan | 2 | 0,44 |
| Libya | 2 | 0,44 |
| Norway | 2 | 0,44 |
| Palestine | 2 | 0,44 |
| South Africa | 2 | 0,44 |
| The Netherlands | 2 | 0,44 |
| Ukraine | 2 | 0,44 |
| Bahrain | 1 | 0,22 |
| Bangladesh | 1 | 0,22 |
| Benin | 1 | 0,22 |
| Bulgaria | 1 | 0,22 |
| Chile | 1 | 0,22 |
| Democratic Republic of The Congo | 1 | 0,22 |
| Ireland | 1 | 0,22 |
| Ivory Coast | 1 | 0,22 |
| Kenya | 1 | 0,22 |
| Malaysia | 1 | 0,22 |
| Mauritania | 1 | 0,22 |
| Moldova | 1 | 0,22 |
| Mozambique | 1 | 0,22 |
| Nigeria | 1 | 0,22 |
| Paraguay | 1 | 0,22 |
| Qatar | 1 | 0,22 |
| Saudi Arabia | 1 | 0,22 |
| Senegal | 1 | 0,22 |
| Serbia | 1 | 0,22 |
| Taiwan | 1 | 0,22 |
| Togo | 1 | 0,22 |
| United Arab Emirates | 1 | 0,22 |
