# Supplementary material for: Artificial intelligence in rheumatology and paediatric rheumatology: insights from an international survey by EMEUNET
Source: EULAR Rheumatol Open. 2026 Apr 3;2(2):100153. doi: 10.1016/j.ero.2026.03.001 (PMC13425164; doi:10.1016/j.ero.2026.03.001)
Supplement: Supplementary file 7 [file mmc7.docx]

**Supplementary Material S5.** AI implementation in the workplace, by region

|  | **Africa N = 102***^1^* | **Asia N = 71***^1^* | **Europe N = 170***^1^* | **North America N = 76***^1^* | **South America N = 36***^1^* |
| --- | --- | --- | --- | --- | --- |
| **In 2025, do you know if your workplace has explored, implemented, or considered using artificial intelligence in clinical practice or research?** |  |  |  |  |  |
| Yes (one or more AI tools have already been implemented) | 13 (13%) | 11 (15%) | 34 (20%) | 36 (47%) | 8 (22%) |
| Yes (one or more AI tools have already been implemented and are actively used in clinical practice and/or research **OR** pilot studies or exploratory projects have been conducted to assess its feasibility and usefulness **OR** pilot studies or exploratory projects have been conducted to assess its feasibility and usefulness) | 25 (25%) | 20 (28%) | 81 (48%) | 59 (78%) | 17 (47%) |
| *^1^* n (%) | | | | | |

Abbreviations: artificial intelligence (AI)
