# Supplementary material for: Artificial intelligence in rheumatology and paediatric rheumatology: insights from an international survey by EMEUNET
Source: EULAR Rheumatol Open. 2026 Apr 3;2(2):100153. doi: 10.1016/j.ero.2026.03.001 (PMC13425164; doi:10.1016/j.ero.2026.03.001)
Supplement: Supplementary file 8 [file mmc8.docx]

**Supplementary Material S6**. Opinions, knowledge, and practical skills by participant category.

| **Characteristics** | **HPRs N = 38***^1^* | **Other N = 14***^1^* | **Physician N = 409***^1^* |
| --- | --- | --- | --- |
| **How important do you think it is for health professionals to have AI literacy?** |  |  |  |
| Extremely important: AI literacy is essential for all health professionals, both now and in the future | 15 (39%) | 7 (50%) | 147 (36%) |
| Moderately important: AI literacy is helpful but not necessarily essential for all health professionals | 4 (11%) | 2 (14%) | 87 (21%) |
| Unsure: I am uncertain about the importance of AI literacy for health professionals | 0 (0%) | 0 (0%) | 6 (1.5%) |
| Very important: AI literacy is becoming increasingly crucial for health professionals | 19 (50%) | 5 (36%) | 168 (41%) |
| **How do you assess the effectiveness of AI tools in your practice? (if other, please specify)** |  |  |  |
| By time saved in routine tasks | 10 (26%) | 6 (43%) | 135 (33%) |
| Do not use AI | 0 (0%) | 0 (0%) | 2 (0.5%) |
| I don’t assess the effectiveness | 13 (34%) | 1 (7.1%) | 103 (25%) |
| Not applicable | 0 (0%) | 0 (0%) | 1 (0.2%) |
| Through personal observation or feedback from colleagues or patients | 11 (29%) | 6 (43%) | 137 (34%) |
| Using specific performance metrics or outcomes | 4 (11%) | 1 (7.1%) | 28 (6.9%) |
| **How would you rate your opinion about AI efficacy and safety?** |  |  |  |
| 1 – Negative or critical | 1 (2.6%) | 0 (0%) | 1 (0.2%) |
| 2 – Skeptical or concerned | 6 (16%) | 0 (0%) | 17 (4.2%) |
| 3 – Neutral or undecided | 9 (24%) | 4 (29%) | 56 (14%) |
| 4 –Optimistic with caution | 19 (50%) | 7 (50%) | 248 (61%) |
| 5 – Positive and optimistic | 3 (7.9%) | 3 (21%) | 86 (21%) |
| **How would you rate your knowledge of AI concepts and applications?** |  |  |  |
| 1 – I have no knowledge or experience | 0 (0%) | 0 (0%) | 43 (11%) |
| 2 – I have basic knowledge with limited experience | 17 (47%) | 5 (36%) | 142 (35%) |
| 3 – I have moderate knowledge but still learning | 13 (36%) | 2 (14%) | 174 (43%) |
| 4 – I have strong knowledge and familiarity with key concepts | 5 (14%) | 4 (29%) | 41 (10%) |
| 5 – I have expert-level knowledge and deep understanding | 1 (2.8%) | 3 (21%) | 9 (2.2%) |
| **How would you rate your practical skills in using AI tools and techniques in AI?** |  |  |  |
| 1 – I have no skills or experience | 4 (11%) | 0 (0%) | 59 (14%) |
| 2 – I have basic skills with limited hands-on experience | 13 (34%) | 6 (43%) | 132 (32%) |
| 3 – I have some skills and experience but still developing | 17 (45%) | 2 (14%) | 176 (43%) |
| 4 – I have solid skills and regular practical experience with AI tools | 3 (7.9%) | 3 (21%) | 36 (8.8%) |
| 5 – I have expert-level skills and extensive experience with AI tools | 1 (2.6%) | 3 (21%) | 6 (1.5%) |

*^1^* n (%) Abbreviations: artificial intelligence (AI), health professionals in rheumatology (HPRs)
